# In suspicious for malignancy thyroid nodule aspirates, nuclei characteristics deserves special attention in reported cytology analysis - real world scenario cohort

**DOI:** 10.20945/2359-4292-2024-0481

**Published:** 2025-09-24

**Authors:** Fabiane Carvalho Macedo, Ricardo Luiz Costantin Delfim, Fernanda Vaisman

**Affiliations:** 1 Endocrinologia, Faculdade de Medicina, Universidade Federal do Rio de Janeiro, Rio de Janeiro, Brazil; 2 Instituto Nacional de Câncer, Rio de Janeiro, RJ, Brazil; 3 DASA; 4 Private Practice, Rio de Janeiro, RJ, Brazil

**Keywords:** Thyroid nodule, Thyroid Neoplasms, Biopsy, fine-needle

## Abstract

**Objective:**

To identify cytologic characteristics in a suspicious for malignancy cohort that may help to recognize false positives in cytopathological tests of thyroid nodules in a “real world scenario”, with histopathological reports as the gold standard.

**Methods:**

Cytomorphologic features of suspicious for malignancy thyroid nodules in a 13-year retrospective database were reviewed. Therefore, we identified false positive cases, analyzed the possible causes of cytopathological diagnostic failure and calculated the frequency of false positive results and the risk of malignancy in the suspicious for malignancy cohort.

**Results:**

Among the 289 suspicious for malignancy type nodules, 283 were malignant, 5 were benign, and 1 was a noninvasive follicular thyroid neoplasm with papillary-like nuclear features (NIFTP). The most frequently reported cytology features were nuclear grooves and pseudoinclusions; however, they were present in malignant and benign specimens. Statistical analysis revealed that the presence of micronucleoli (p < 0.001) and/or irregular/oval nuclei (p = 0.05) were the characteristics most strongly associated with malignancy. The risk of malignancy was 98% in this study.

**Conclusion:**

The presence of micronucleoli and nuclear irregularity was highly predictive of malignancy according to suspicious for malignancy cytology and were not present in false positive patients. Hence, careful examination of nuclear characteristics can be helpful for identifying true malignancies via suspicious for malignancy cytology. This was significant even when only a qualitative analysis was taken into account.

## INTRODUCTION

Papillary thyroid carcinoma (PTC) and follicular carcinoma constitute differentiated thyroid carcinomas (DTC) and account for more than 90% of thyroid cancer cases (^1^). Statistical data showed that in 2023, the total estimated number of new thyroid cancer cases in the United States, in both sexes, was 43,720, the majority of which were women (31,180 cases) (^2^). In Brazil, where the incidence shows the same tendency to primarily affect women, data from the National Cancer Institute (INCA) showed an estimated 14,160 cases (5.8%) of thyroid cancer in women in 2023, making it the fifth most common malignant neoplasm in this group (^3^). Among DTC, PTC is the most common subtype (^4^,^5^) and is responsible for 80 to 85% of thyroid cancer cases in the United States (^5^).

Fine-needle aspiration biopsy (FNAB) has proven to be a reliable method for assessing thyroid tumors and is a safe, minimally invasive and cost-effective technique. With a high level of accuracy in predicting malignancy, especially papillary carcinoma, in satisfactory specimens, the diagnostic accuracy can reach 95%, with positive predictive values ranging from 89 to 98%. In addition, with an overall sensitivity of 83 to 92% for providing a definitive diagnosis and a specificity ranging from 75 to 97%, as reported in several publications (^6^-^8^), this approach is crucial for patients with thyroid tumors. This technique helps with both timely screening for available surgical interventions and avoiding unnecessary surgery in patients with benign nodules (^9^,^10^). However, similar to any diagnostic method, FNAB can generate false-negative and false positive results.

The suspicious for malignancy (SFM) category in the publication The Bethesda System for Reporting Thyroid Cytopathology (TBSRTC), the most recent review from 2023, third edition, is associated with a mean risk of malignancy (ROM) of 74% (^9^). In this publication, as well as in the second edition (^11^), 16 cytomorphological criteria are listed that can be observed in papillary carcinoma, classic or conventional variants and any combination between them, but the quantity, importance, and frequency of each of them are undefined (^9^,^11^).

Previous studies have shown that malignancy rates in SFM category are widely variable, ranging from 51% (^12^) to 96% (^13^) or 100% (^14^-^17^). The recommended approach for SFM category is surgery, molecular testing, lobectomy, or near-total thyroidectomy (^9^). Nevertheless, molecular tests have shown divergent results, with some studies like Talmor and cols. showing that a particular molecular test (Afirma GEC molecular test) was sensitive but not very specific for the final histologic diagnosis of cancer, and that a suspicious test result was not a significant predictor of malignancy (^15^). Figge and cols. using a different molecular test (ThyroSeq V3) have shown high sensitivity and reasonably high specificity for classifying indeterminate thyroid nodules as benign or malignant (^18^). Therefore, filtering out false positive cases, although rare, is challenging.

Due to the great heterogeneity of diagnoses in the SFM category, as well as the wide range of ROMs in this category, and the lack of Brazilian data in the literature, the study of possible cytological characteristics that influence the diagnostic accuracy of the SFM category is relevant.

Thus, our objective was to identify cytologic characteristics in a SFM cohort that may help to recognize false positives in cytopathological tests of thyroid nodules in a “real world scenario”, with histopathological reports as the gold standard.

## METHODS

### Study design

This was an observational study with a retrospective phase of data review in the form of cytopathological, histopathological and ultrasound (US) reports, printed and/or digital, in a selected group of patients. This study was approved by the Ethic Committee (no. 0535560/12), and the need for free and informed consent was waived (Certificate of Presentation for Ethical Appreciation number 02266912.6.0000.5257).

This study was conducted based on the identification of a group of 336 patient nodules with SFM cytology and its subcategories that had histopathological reports, from a database of a specialized radiologist dedicated to the performance of US-guided FNABs. The American College of Radiology Thyroid Imaging Reporting and Data System (ACR TI-RADS) was chosen as the standard US rating classification. Although hypoechoic nodules have been classified into mild, moderate and marked ones, the two first ones were considered as hypoechoic nodules, as rated by the ACR TI-RADS (^19^).

The database had 8,596 thyroid nodules that underwent FNAB: 4,548 (53%) were benign, 1,734 (20%) nondiagnostic, 1,102 (13%) atypia of undetermined significance, 210 (2%) follicular neoplasia, 650 (8%) SFM and 352 (4%) malignant. Of the 650 SFM nodules, 314 patients who had lesions were lost during follow-up. Thirty-nine nodules were excluded because the cytological and/or histopathological reports were not retrievable, and eight patients were excluded because they were in the pediatric group (under 20 years old), which was not the target of the study. All the FNABs were carried out between 2008 and 2021 and followed a protocol previously described using 22 g needles attached to 20 mL syringes (^20^) The aspirated material was placed on slides and fixed in 95% alcohol. After that, the residual aspirate was fixed in 10% formalin and paraffin-embedded (cell block). The aspirates were sent to different laboratories throughout the study; conventional smear slides were stained using the Papanicolaou technique, and the cell blocks were stained using the hematoxylin-eosin (HE) technique. All the slides were examined under light microscopy by several cytopathologists, sometimes more than one cytopathologist, in accordance with each laboratory’s standards. The cell blocks were examined as a complementary diagnostic tool. Most surgical specimens resulting from total thyroidectomies were processed according to standard surgical pathology procedures, generating HE-stained slides that were finally analyzed by pathologists specializing in the field via optical microscopy.

The cytological and histopathological reports, printed or digital, were carefully analyzed by an experienced pathologist who reviewed the cytopathology reports to determine which of the sixteen characteristics listed in the TBSRTC for diagnosing PTC were being mentioned and ultimately yielded the diagnosis of SFM. The data were entered into a spreadsheet for each nodule as present or absent for all types of features. The final diagnosis, size of nodules, PTC subtype and multifocality data were collected from the histopathological reports and matched with the US and cytological information of nodules previously examined and aspirated. The demographic and US characteristics were entered into the spreadsheet by the radiologist.

Therefore, it was identified cases with divergent cytopathological and histopathological results, that is, false positive cases that were classified as SFM on cytopathology and after thyroid surgery that received a histopathological result of a benign thyroid disease or noninvasive follicular neoplasia with papillary-like nuclear features (NIFTP). From there, we analyzed the possible causes of diagnostic failure in cytopathological examination, calculated the frequency of false positivity and the ROM for the SFM category, with histopathological results as the gold standard, and finally analyzed the statistical significance in the cohort of each cytological criterion cited in the TBSRTC for the diagnosis of PTC.

### Statistical analysis

Descriptive statistics resources were used to present the data in tables of frequencies or proportions and other forms of data. Continuous variables are presented as the means ± standard deviations or medians (interquartile ranges), while categorical variables are presented as proportions. When relevant, variables were compared between groups using the chi-square test or Fisher’s exact test when the distribution was for non-normal distributions and variables were categorical, Mann-Whitney test, Student’s *t* test data follows a normal distribution and were numeric for two groups or analysis of variance (Anova) for multiple groups, depending on the desired clinical interpretation and the behavior of the variables. Common normality tests used was Kolmogorov-Smirnov test. The Mann-Whitney U test is used to compare differences between two independent groups when the dependent variable is either ordinal or continuous, but not normally distributed.

The significance level was always set at 5%, and 95% confidence intervals were calculated whenever applicable. To assess the relationship between variables and the confirmation of postoperative malignancy, a stepwise logistic regression model was used, adjusting for other potential confounding factors. Only variables strongly associated with the presence of malignancy in the univariate analysis (p < 0.05) were included in the final model to perform the multivariate analysis and hazard ratio. All the statistical analyses were performed using SPSS version 20.0 for MAC.

## RESULTS

Results were based on a review of 289 SFM nodules from 271 adult patients (≥ 20 years old). In this series, 185 of 188 nodules were carcinomas (ROM: 98%) before the 2017 Bethesda revision, and 98 of 101 were carcinomas (ROM: 97%) after it.

In our cohort of 289 SFM nodules, 283 (98%) were in fact diagnosed as malignant on histopathological examination. Among the five nodules (1.7%), the diagnosis was benign thyroid disease, and one nodule (0.3%) was diagnosed as noninvasive follicular NIFTP. Among the 283 truly malignant cases, 274 belonged to the Bethesda subcategory suspicious for papillary carcinoma, and nine nodules from seven patients were classified in the subcategory suspicious for medullary carcinoma. In six of these seven patients, the histopathology results agreed with the cytopathology subcategory, and only one patient received a PTC diagnosis in the final histopathological report. There was agreement between histology and cytology in six of seven patients with a final histopathology result of medullary thyroid carcinoma, with only one patient previously classified in the Bethesda subcategory suspicious for papillary carcinoma.

**Table 1** displays all the general characteristics of the patients and ultrasonographic features of the nodules. Among the 271 patients with 289 nodules, 240 patients (83%) were female, 49 patients (17%) were male, and the median age was 45 years, ranging from 20-83 years. Nodules were between 1.01 and 2.0 cm in 45.7% (132/289) of patients, and in our cohort, nodules smaller than 1.0 cm were also aspirated, representing 37.7% (109/289) of patients; however, currently, this approach is not recommended by endocrinology societies. This was partly because we had many nodules that were aspirated before this recommendation was established in the thyroid consensuses. Besides that, all patients were referred to FNAB by their attending physician’s clinical management, including those presenting sub-centimeter nodules, in outpatient settings. Whilst ultrasound analysis and TI-RADS rating were conducted by the ultrasound guided procedure’s performing physician.

**Table 1:** General characteristics of patients (271) and nodules (289)

		N	%	Mean (SD) Median (min. max.)
Age at assessment (years)	20-35 36-55 56-75 76-85	68 132 65 06	25.1 48.7 24.0 2.2	45.85 (13.79) 45 (20-83 y)
Gender F:M		240:49	83:17	
USG Characteristics of Nodules		N	%	
**Tumor size (cm)**	≤ 1.0 1.01-2.0 2.01-4.0 ≥ 4.01	109 132 46 02	37.7 45.7 15.9 0.7	1.0 (0.765) 1.2 (0.4-6.7 cm)
Echogenicity Hyperechoic Isoechoic Hypoechoic Mild Moderate Marked		02 42 245 67 99 79	0.7 14.5 84.8 27.3 40.4 32.2	
Nature Solid Mixed Cystic		281 03 05	97.2 1.0 1.8	
Margin Regular Irregular		110 179	38.1 61.9	
Hyperechogenic foci^*^ Microcalcification Macrocalcification Colloid crystals Unspecific hyperechoic spots Peripheral calcification Absent		111 36 05 36 10 121	38.4 12.5 1.7 12.5 3.5 41.9	
ACR TI-RADS 1 2 3 4 5		00 07 04 81 197	0 2.4 1.4 28.0 68.2	

* For reference, see (^19^). It is possible to have more than one feature in the same nodule.

USG: ultrasonographic; ACR TI-RADS: American College of Radiology Thyroid Imaging Reporting and Data System.

On US, the thyroid volume was normal in 256 patients (88.6%) and increased in only 30 patients (10.4%). Previous FNAB results were reported for 23/278 patients (8.3%), with atypia of undetermined significance and nondiagnostic being the most frequent classifications for 10 patients (43.5%) and 8 patients (34.8%), respectively.

Thyroid function was normal in 247 patients (85.5%) based on laboratory tests; hypothyroidism was observed in 37 (12.8%) patients, and hyperthyroidism was detected in five patients (1.7%).

There were 55 patients (19%) with chronic thyroiditis according to US or laboratory tests; of these, 4/55 patients were reported as having associated chronic thyroiditis on cytology as well as 7 patients on histopathological examination.

Hypoechogenicity was observed in 245 patients (84.8%), isoechogenicity was in 42, and hyperechogenicity was observed in 2 patients. Hypoechogenicity was then subdivided into three degrees according to a previous study carried out by this group: mild, moderate and marked hypoechogenicity. Sixty-seven (27.3%) nodules were considered to show mild hypoechogenicity, 99 (40.4%) moderate hypoechogenicity, and 79 (32.2%) marked hypoechogenicity. Among the false positive patients, three had nodules showing isoechogenic features, and one of each had mild, moderate or marked hypoechogenicity (**Table 2**).

**Table 2:** Characteristics of false positives

Case no	Age	Sex	Size on US	TI-RADS	PrevioUS FNAB	Echogenicity	Composition	Pathology report	Size on path report
1	41	F	1.47	2	--	Isoechoic	Predom solid	NH	1.3
2	43	F	1.6	4	--	Isoechoic	Solid	Nodular Thy	0
3	58	F	1.6	4	--	Mild hypoechoic	Solid	FA	1.5
4	68	F	6.3	4	--	Moderate hypoechoic	Solid	Hashimoto´s Thy	0
5	52	F	1.1	4	--	Marked hypoechoic	Solid	NIFTP	1.0
6	64	F	2.3	4	3	Isoechoic	Solid	OCH	3.5

USG: ultrasonographic; TI-RADS: Thyroid Imaging Reporting and Data System; FNAB: fine-needle aspiration biopsy; F: female; NH: nodular hyperplasia; Thy: thyroiditis; FA: follicular adenoma; NIFTP: noninvasive follicular thyroid neoplasm with papillary-like nuclear features; OCH: oncocytic cell hyperplasia.

In terms of composition, the vast majority of nodules were solid (259; 89.6%) or predominantly solid (22; 7.6%). Among the false positive patients, five had solid nodules, and one had a predominantly solid nodule. Most nodules (197; 68.2%) were classified as TI-RADS 5, whereas among the false positive patients, five were TI-RADS 4, and one was TI-RADS 2.

The sixteen cytomorphological criteria that were analyzed are shown in **Table 3** and included criteria such as the presence of grooves, pseudoinclusions, irregular or oval nuclei, the presence of micronucleoli, and a papillary or monolayer configuration, among others.

**Table 3:** Frequency of cyto-morphological features of suspicious for malignancy nodules

	n (%)	%
Papillary and/or monolayer arrangement	153 (52.9)	
Nuclear enlargement and overlap	91 (31.5)	
Irregular or oval nuclei	209 (72.3)	
Grooves	269 (93.1)	
Pseudoinclusions	221 (76.5)	
Nuclear clearing	62 (21.5)	
Micronucleoli	80 (27.7)	
Psammoma bodies	23 (7.9)	
Multinucleated cells	79 (27.3)	
Swirls/cartwheel patterns	0	0
Presence of colloid	103 (35.6)	
Thick nuclear membranes	2 (0.7)	
Oncocytic metaplasia	37 (12.8)	
Hobnail cells	0	0
Squamous metaplasia	1 (0,^3^)	
Histiocytoid cells	1 (0,^3^)	
Associated lymphocytic thyroiditis	4 (1.4)	

The cytological feature that most frequently appeared in cytopathological reports was the presence of nuclear grooves (269 patients, 93.1%), followed by pseudoinclusions (221 patients, 76.5%), irregular or oval nuclei (209 patients, 72.3%), and papillary and/or monolayer arrangement (153 patients, 52.9%). However, colloid substances were mentioned in 103 reports (35.6%) of the 289 nodules, followed by nuclear enlargement and overlap (91 cases, 31.5%), the presence of micronucleoli (80 cases, 27.7%) and the presence of multinucleated cells (79 cases, 27.3%). The cytopathological reports did not provide details regarding the type or appearance of the colloid observed. Nuclear clearing was referred to in only 62 (21.5%) thyroid nodule reports. Other characteristics, such as oncocytic metaplasia (37 patients, 12.8%), psammoma body identification (23 patients, 7.9%), thickening of the nuclear membrane (2 patients, 0.7%), and presence of squamous metaplasia (1 patient, 0,3%) were mentioned very rarely in the reports. Finally, the swirls/cartwheel patterns, hobnail and histiocytoid appearance of follicular cells were not described in any of the reports.

Associated lymphocytic thyroiditis was referred to in the cytopathological reports of only 4 FNAB patients in our cohort (1.4%); none of these four patients were in the false positive group.

**Figure 1:**
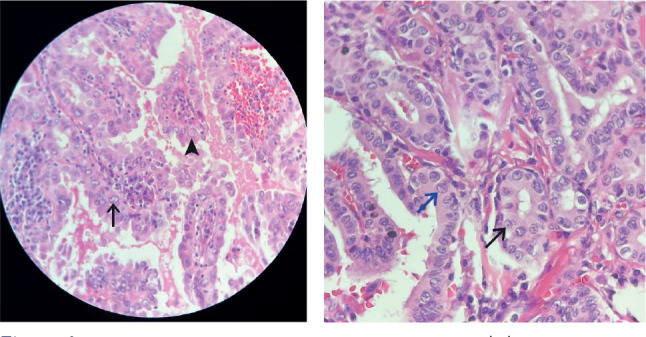
Papillary thyroid carcinoma case showing (A) hematoxylin-eosin 100x, papillary fronds lined by cells with irregular nuclei, prominent micronucleoli (black arrow) and pseudoinclusion (arrowhead); (B) hematoxylin-eosin 400x, enlarged and irregular nuclei with prominent micronucleoli (black arrow) and grooves (blue arrow).

**Figure 2:**
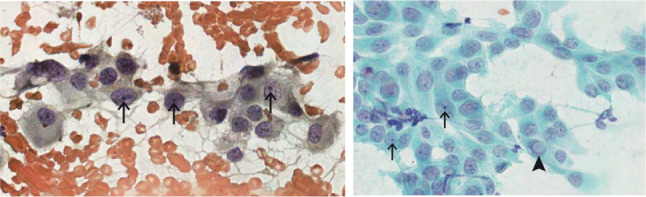
(A) Papanicolaou 400x. Cells with prominent micronucleoli (black arrows) in oval/irregular nuclei. (B) Papanicolaou 400x. Cell group with irregular and enlarged nuclei showing prominent micronucleoli (black arrows) and a pseudoinclusion (arrowhead).

Statistical analysis revealed that although they did not appear most often in cytopathological reports, the presence of micronucleoli (p < 0.001) and irregular or oval nuclei (p = 0.05) were the characteristics most strongly associated with malignancy. The presence of grooves, pseudoinclusions, nuclear enlargement, papillary arrangement and nuclear clearing are the characteristics most often cited in the cytopathology literature and in the routine practice of cytopathology laboratories as essential criteria for the diagnosis of PTC. However, these parameters did not significantly predict malignancy in our cohort (**Table 4**). **Figure 1** shows an example of papillary thyroid cancer and **Figure 2** shows the nuclei alterations that were statistically significant to predict malignancy.

**Table 4:** Association of cytological features and malignancy

	Malignant = 283	Benign = 6	p-value	HR	p-value
Papillary and/or monolayer arrangement	151	2	0.710		
Nuclear enlargement and overlap	87	4	0.210		
Irregular or oval nuclei	207	2	**0.018**	4.9 (1.2-26.2)	0.050
Grooves	264	5	0.390		
Pseudoinclusions	217	4	0.660		
Nuclear Clearing	61	1	1.000		
Micronucleoli	80	0	**0.030**	13.8 (7-20.9)	<0.001
Psammoma bodies	23	0	0.440		
Multinucleated Cells	77	2	0.940		
Swirls/cartwheel patterns	0	0	1.000		
Presence of colloid	102	1	0.690		
Thick nuclear membranes	2	0	1.000		
Oncocytic metaplasia	37	0	0.600		
Hobnail cells	0	0	1.000		
Squamous metaplasia	1	0	0.870		
Histiocytoid Cells	0	0	1.000		
Associated lymphocytic thyroiditis	4	0	**0.090**		

HR: hazard ratio.

## DISCUSSION

In the present study, with a large Brazilian cohort of SFM nodules, both the presence of micronucleoli and the presence of oval/irregular nuclei were the two cytological characteristics that were highly predictive of malignancy and were also found to be independent predictors; when present, they can increase the chance of malignancy nearly 5- and 13-fold.

It is already well known that nuclear changes are very important features in the diagnosis of thyroid carcinoma, especially PTC. Regardless of the PTC subtype, cytopathology and histopathology are essential for diagnosis. Morphological changes are complementary and can occur in some subtypes, such as the papillary arrangement in the classic subtype; on other thyroid tumors, they are even dispensable, as in follicular variants, where only a follicular arrangement is present rather than a papillary arrangement. In fact, there are six nuclear criteria recommended by the TBSRTC as the minimum requirement for diagnosing malignancy (enlarged nuclei, longitudinal nuclear grooves, intranuclear pseudoinclusions, pale nuclei with powdery chromatin, marginally placed micronucleoli and oval or irregularly shaped nuclei) (^9^,^21^,^22^). Notably, the presence of micronucleoli and oval/irregular nuclei were found to be statistically significant in our study. Other nuclear characteristics have been shown to be important in determining true malignant specimens among SFM nodule samples. Ohori and cols. (^22^) reported that ≥ 3 intranuclear pseudoinclusions is the best criterion for discriminating Malignant from SFM Bethesda category. In our study, as in others like Higgins and cols. (^12^), the presence of nuclear pseudoinclusions was very frequent, as reported in 217/283 of our histologically proven malignant cytological reports but also in 4/6 histologically proven benign cytological reports, resulting in no statistical relevance. It therefore remains to be investigated which set of criteria should be used and how many should be minimally required for the diagnosis of carcinoma, especially PTC, to minimize false positivity.

Quantitative analysis was not performed in our study as only the reports were reviewed instead of the slides. However, even in these qualitative analyses, the presence of these two nuclei alterations, especially the presence of micronucleoli were highly predictive of malignancy (increasing the risk by almost 14 times). Maybe, in the presence of that, cytology should be considered Malignant, instead of SFM.

There are also reports of an association between these nuclear abnormalities and certain molecular alterations, as in the study by Recupero and cols. (^23^) They considered that these nuclear alterations in PTC are a combination of altered distribution of chromatin and irregularities in the shape of the nuclear membrane and investigated the hypothesis that these events are related to the overexpression of the lamin B receptor, a supportive protein located on the inner face of the nuclear membrane, which is responsible for the distribution of lamin B and the associated chromatin (^23^).

Studies investigating whether nuclear inclusions play a biological role in tumorigenesis have shown that intranuclear inclusions are invaginations of the cytoplasm that have completely closed off the nucleus and lost their connection to the cytoplasm; these inclusions contain proteins associated with autophagy, degenerated organelles and lysosome-like proteases. These findings suggest that these inclusions play a role in autophagy and proteolysis. It has also been shown that the number of these nuclear inclusions is positively associated with the BRAFV600E mutation and that mutant BRAF is present within these inclusions and colocalizes with proteins associated with autophagy, exposing them to the proteolytic process (^24^).

Papanicolaou technique is the standard staining used by local pathology laboratories and was the technique used in all specimens in the study. On the other hand, HE and panoptic stains are alternatives based on pathologist preferences. In addition, panoptic stain requires a dry smear, unlike Papanicolaou and HE.

Regarding the cytomorphological characteristics mentioned here in the false positive cases, grooves were mentioned in five out of six cases; nuclear enlargement and pseudoinclusion were mentioned in four out six cases; papillary arrangement, irregular or oval nuclei and multinucleated cells were mentioned in two out of six cases; and nuclear clearing and the presence of colloids were mentioned in one out of six cases. Several studies and case reviews have shown that fragments of papillary-like structures (without central cores) can occur in chronic lymphocytic thyroiditis and nodular hyperplasia; these fragments are papillary clusters, sometimes in a honeycomb pattern with well-defined borders and evenly distributed cells, simulating true papillae that have central cores and a syncytial arrangement of unevenly distributed cells with nuclear crowding, overlapping or molding (^21^,^25^-^27^). Cytological features such as intranuclear grooves, pseudoinclusions, nuclear enlargement, nuclear clearing and nuclear membrane irregularity are observed in cytological samples of several different benign thyroid diseases, such as the ones that appeared in the final pathological reports in this study: nodular hyperplasia, chronic lymphocytic thyroiditis, follicular adenoma and oncocytic lesions.

In our study and in the literature, intranuclear grooves and pseudoinclusions are the two cytological features most frequently observed in patients with these mimicker diseases (^9^,^21^,^25^-^29^). Care must be taken not to misdiagnose mimickers or incomplete or sparse features such as PTC. For this purpose, it must be ensured that exams are carried out by well-trained professionals in both radiology and cytopathology, with constant updating of their knowledge and practices. Similarly, well-represented samples with adequate cellularity, appropriate processing and staining, and strict adherence to diagnostic criteria are important for reducing false positivity rates (^21^).

Like other series in literature (^30^), indeterminate categories (Atypia of undetermined significance, Follicular neoplasia and SFM) represented around 20% of all FNABs in our study. The malignancy rates for SFM category in thyroid nodules have been reported to be higher than initially expected in “real world scenario” studies. According to a study analyzing data from the National Surgical Quality Improvement Program (NSQIP), the malignancy rate for SFM thyroid nodules was found to be 91.1% (^33^). This is significantly higher than the 52.5% malignancy rate reported in the 2017 TBSRTC.11

Another multicenter study conducted in Israel reported a malignancy rate of 86.9% for SFM nodules (^32^). Similarly, a single-center study outside the United States found a malignancy rate of 91.3% for this category (^33^).

In addition, our cohort showed, in a real-world scenario, where only reports of the 289 specimens were analyzed, 98% of malignancy. Likewise, the false positive rate of 2% calculated in our SFM cytopathology cohort is similar to that reported in the literature, in which a range of 2 to 10% has been reported for FNABs (^21^,^25^,^34^-^36^).

In a South Korean study by Yi et al.,^31^ the false positive rate for FNABs was even lower (1.4%), but this study included a much larger number of thyroidectomies and included 14 Malignant cases, a category with a higher positive predictive value, in the analysis, in addition to the 34 SFM cases. In another 2011 study, Lew and cols. (^34^) reported a 2% (3/147) false positive rate in a group of patients who had undergone thyroidectomy prior to the introduction of the TBSRTC but whose cytology had already been classified as SFM or malignant, which would currently correspond to SFM and Malignant categories, respectively. In this study, Lew et al. considered only the malignant category to calculate the false positive rate, omitting the SFM one (^34^). However, the figures are explicit, and if we calculate the false positive rate for the SFM category only, it would be 8% (7/85), which is within the range reported in most studies.

The ROM of 98% that we observed in our study for SFM category was much greater than the average of 74% (NIFTP = cancer) or 65% (NIFTP ≠ cancer) reported in the latest edition of TBSRTC.9 In previous similar studies, values ranging from 51% (^12^) to 96% (^13^) or 100% (^14^-^17^) were found, and in all these studies, the number of individuals involved (nodules or patients) was much smaller than that in our study. The study that achieved 100% ROM included only 8 SFM patients (^14^). A Brazilian case series from 2018 analyzed the Bethesda classification in a reference center for the treatment of thyroid diseases and reported a ROM of 72.5% in 40 patients classified as SFM category who subsequently underwent thyroidectomy (^17^). The vast majority of these studies not only examined the SFM category but also analyzed the ROM of all Bethesda categories or only the so-called indeterminate categories (Atypia of undetermined significance, Follicular neoplasia and SFM). To the best of our knowledge, our cohort is the most extensive SFM nodules surgery case series to date. Additionally, the ROM of 98% in those properly selected nodules would dismiss the need for further investigation before surgery, such as molecular testing, to define the extent of surgery, as recommended by some authors (^18^,^37^,^38^).

In TBSRTC it is stated that “a distinction between a malignant and suspicious diagnosis (and between suspicious and atypical) is admittedly subjective. A malignant diagnosis should be reserved for those cases that show sufficient cellularity and most, if not all, of the diagnostic features of an entity”. This important issue may be one explanation why some cohorts, including ours, report different ROM for this category. In addition, as this is a real-world study in which cytology was analyzed by different cytopathologists, of which were not all specialized in thyroid, and over a significant period (2008-2021), in a situation of diagnostic uncertainty, those tend to consider that the characteristics are not “sufficiently” represented and report as SFM category instead of the Malignant one, in order to keep the very high positive predictive. Furthermore, until a few years ago, there was no data about NIFTP and some of our true malignant cases, before that entity was described, could have been misclassified.

Of the six patients with false positive cytopathological results in the SFM cohort identified in our study, five were subcategorized as suspicious for PTC, and one was subcategorized as suspicious for lymphoma. The final histopathological results of these six patients were as follows: nodular hyperplasia (one case), chronic thyroiditis (two cases), follicular adenoma (one case), NIFTP (one case) and oncocytic cell hyperplasia (one case). One of the patients with histopathological proven chronic lymphocytic thyroiditis (Hashimoto’s) was the only patient in the cohort classified as SFM/suspicious for lymphoma. However, this predictable diagnostic failure can occur since these two entities are differential diagnoses of each other, despite the cytological specimens being analyzed by pathologists from two renowned centers before surgery.

Chronic lymphocytic thyroiditis is often listed as one of the conditions producing some of the nuclear characteristics of PTC and frequently causes false positive results for SFM and malignant categories in cytopathological exams (^6^,^7^,^9^,^25^,^28^,^38^). Therefore, we took care to note the referral to that diagnosis in the US and laboratory tests (55 patients, 19%), as well as in cytopathological and histopathological reports, which occurred in only 4 (1.4%) and 7 (2.4%) patients, respectively, in our cohort. None of these patients were in the false positive group, leading us to conclude that in our cohort, thyroiditis was not an important cause of false positivity.

Regarding false positive cases with a histopathological diagnosis of oncocytic cell hyperplasia, the oncocytic cell itself may have nuclear features that can be misinterpreted as diagnostic criteria for papillary carcinoma (grooves, increased nuclear size, macronucleoli and nuclear pallor) (^39^,^40^). In addition, this patient was the only false positive patient who had previously undergone aspiration and given a cytopathological diagnosis of Atypia of undetermined significance. It is possible that, in addition to the alterations typical of oncocytic cells, in this case, we also had regenerative or reparative alterations due to the multiple passes of the needle in the previous procedure (^25^). After an FNAB procedure, fibrosis, hemorrhage, necrosis, vascular and/or capsular pseudoinvasion, cholesterol granulomas, and nuclear atypia, such as nuclear enlargement, as well as grooves and pseudoinclusions, can be detected, even incomplete or suboptimal, which are the confounding factors in false positive cytopathological diagnosis (^25^,^26^).

The other four false positive cytopathological cases were diagnosed on histopathology as nodular hyperplasia, chronic lymphocytic thyroiditis, follicular adenoma and NIFTP, all of which have been widely studied and cited in the literature as potential mimickers of the diagnostic criteria for PTC (^26^,^27^). In this study, we considered the only NIFTP case to be a false positive diagnosis since these lesions are regarded as *very low risk malignancies* with an exceedingly indolent behavior, despite sharing some cytological findings with PTC (^41^-^44^). One possibility for this paucity of NIFTP diagnoses is that our data collection began 8 years before the diagnosis of NIFTP was formalized (^41^). Another possibility could be the close observance of the NIFTP diagnostic criteria by pathologists in the pathology reports of resection specimens. Therefore, the scarcity of this diagnosis in our series had no effect on the ROM of the analyzed SFM category (^21^,^22^). Nevertheless, this is a controversial subject. Several articles have shown that malignancy rates decrease when NIFTP is considered nonmalignant, especially for SFM and Malignant categories, with reductions ranging from 9.1% (^9^) to 14% (^45^) or 23.4% (^46^) for SFM category.

In terms of US analysis of false-positive nodules, three of six nodules were isoechoic and one of six nodules was mildly hypoechoic. These data are aligned with the results of other studies showing that mildly hypoechoic nodules are not associated with malignancy, as well as the consistent association of isoechoic and hyperechoic nodules with benign nature (^20^,^47^).

There was a good correspondence between cytology and histology in six of seven patients classified in the subcategory suspicious for medullary carcinoma who received a result of medullary thyroid carcinoma on histology analysis, with only one remaining patient previously classified as suspicious for medullary carcinoma receiving a diagnosis of PTC on histology. From that it can be inferred that although the diagnosis of medullary thyroid carcinoma can be challenging on cytopathology, given its cytologic overlap with other tumors, the group demonstrated good levels of concordance.

Our study has several limitations that should be highlighted. Regarding the US, the operator was a referral specialist with over 25 years of experience in thyroidology who was skilled in minimal procedures, possibly introducing selection bias and favoring the high ROM calculated in our cohort (^18^). However, for such a well-selected set of suspicious nodules, the results from these few false positive cases are even more significant. Regarding cytopathological analysis, FNAB aspirates were sent to several diagnostic centers over the years for cytological examination and were analyzed by different cytopathologists with various training, academic backgrounds and, above all, different levels of experience, even so in our sample, the false positive rate is very low. Interestingly, the results were not significantly impacted by this interobserver variability, and we did not observe any great increase in the dispersion of the diagnostic criteria. In contrast, we observed adherence to the diagnostic criteria, with preservation of the high positive predictive value of the test and apparent maintenance of the degree of subjectivity inherent to the test itself.

Reinforcing these findings, the smallest proportion of false positive results in our exclusive group of SFM patients is comparable to the range reported in the literature for FNABs, generating the highest ROM observed (98%), which is in line with the malignant category ROMs reported by other authors (^9^). Prospective studies with larger sample sizes and multinstitutional case series are required to further investigate our findings.

In conclusion, nuclear characteristics are historically considered a milestone for the diagnosis of PTC. The suspicious for malignancy thyroid cytology category seems to be the key to selecting patients who need surgery with no further investigation. In our study, even qualitative analysis was able to show an important increase in malignancy risk by the presence of micronucleoli and the presence of oval/irregular nuclei. Also, as higher than expected rates of malignancies in the suspicious for malignancy category might be seen around the world, it is important to try to tailor those features that can help less experienced cytopathologists to correctly identify suspicious for malignancy Bethesda category.

## Data Availability

the data that support the findings of this study are available from the corresponding author upon reasonable request.

## Data availability:

datasets related to this article will be available upon request to the corresponding author.
